# Highly Active Palladium-Decorated Reduced Graphene Oxides for Heterogeneous Catalysis and Electrocatalysis: Hydrogen Production from Formaldehyde and Electrochemical Formaldehyde Detection

**DOI:** 10.3390/nano12111890

**Published:** 2022-05-31

**Authors:** Xiaogang Liu, Wenjie Chen, Xin Zhang

**Affiliations:** College of Chemistry and Chemical Engineering, Xinyang Normal University, Xinyang 464000, China; chen2wen9jie5@163.com (W.C.); zhangxin109zz@163.com (X.Z.)

**Keywords:** palladium, graphene, hydrogen production, formaldehyde detection

## Abstract

The exploitation of highly efficient and stable hydrogen generation from chemical storage of formaldehyde (FA) is of great significance to the sustainable development of the future. Moreover, developing an accurate, rapid, reliable, and cost-effective catalyst for electrochemical detection of FA in solution is appealing. Herein, we report rational construction of Pd nanoparticles decorated reduced graphene oxides (Pd/rGO) nanohybrids not only as robust catalysts to produce hydrogen from alkaline FA solution and but also electrocatalysts for electrochemical detection of FA. By optimizing the reaction parameters including FA concentration, NaOH concentration and reaction temperature, Pd/rGO with Pd loading of 0.5 wt% could exhibit a high hydrogen production rate of 272 mL g^−1^min^−1^ at room temperature of 25 °C, which is 3.2 times that of conventional Pd NPs. In addition, as-prepared Pd/rGO nanohybrids modified glassy carbon (GC) electrodes are used as FA-detected electrochemical sensors. A sensitive oxidation peak with a current density of 8.38 mA/cm^2^ was observed at 0.12 V (vs. Ag/AgCl) in 0.5 M NaOH containing 10 mM FA over Pd/rGO catalysts with Pd loading of 0.5 wt%. The results showed the prepared Pd/rGO nanocatalyst not only exhibited efficient and stable hydrogen production from alkaline FA solution but also had good electrocatalytic properties with respect to formaldehyde electrooxidation as a result of the synergistic effect of Pd NPs and rGO nanosheets.

## 1. Introduction

With the rapid development of the world economy and the accompanying increased global warming, the demand for cost-efficient and renewable energy worldwide has increased significantly [1,2]. Hydrogen is generally regarded as a reliable energy alternative to traditional fossil fuels due to its environmental-friendliness, high energy density, and easy polymorphic storage. So far, the mainly hydrogen sources include fossil fuels [3,4], methanol reforming [5], and electrochemical water splitting [6,7,8], which are plagued by low efficiency, high energy consumption, complex operations, and emission of carbon dioxide. However, efficient, low-cost, safe, and selective hydrogen generation from chemical storage materials remains a challenge. To this end, catalytic hydrogen (H_2_) production from inexpensive formaldehyde (HCHO, FA) has aroused wide concern in recent years, as this formaldehyde reforming dehydrogenation reaction (FRR) could release H_2_ at a high theoretical weight of 8.4% [9,10]. To date, both noble metal catalysts such as Pd, Pt, Ag, Au, Ru, and non-noble metal catalysts, such as Ni, and Cu, have been used as efficient catalysts for H_2_ production in FRR [11,12,13,14]. Generally, these active component catalysts are supported on matrix materials to form host–guest materials (supported catalysts), thus ensuring the maximum utilization of reactive sites. Above all, the overall catalytic performance of supported catalysts can be rationally tuned by the noble or non-noble metal particles, as well as supports. Among them, Pd-based catalysts have shown more excellent H_2_ production behavior in FRR. For instance, Pd nanotube was prepared and used as highly efficient hydrogen production from alkaline formaldehyde solution [15]. Although it achieves a relatively higher H_2_ production rate of 778 mmol/(g·h), the high price of used catalysts rendered its further application disadvantageous and made it less competitive. Our previous studies also suggested that Pd nanoparticles (NPs) supported on Fe_2_O_3_ nanoplate, 3D hierarchical porous carbon, and Ti_3_AlC_2_ MAX phase could serve as highly efficient catalysts to produce H_2_ from aqueous formaldehyde solution [16,17,18]. In addition, Pd NPs supported on metal oxide, such as ZnO nanoroads and TiO_2_ nanosheets exposed with (001) facets, realizes efficient H_2_ production from formaldehyde solution [19,20]. However, it is worth mentioning that these Pd-based catalysts with high H_2_ production in FRR have had Pd loading of 1–5 wt%. Such relatively high Pd loadings are sometimes necessary for laboratory research, but for scaled-up production and further applications, it is desirable to have lower Pd loadings (e.g., <1 wt%). Therefore, it would be of great significance to develop a well-defined Pd supported catalyst while preserving its high reactivity, selectivity, and stability for boosting H_2_ evolution from HCHO aqueous reforming.

Apart from its elimination from aqueous solution, given the toxicity of FA, it is crucial to develop accurate, rapid, and reliable FA detection methods, especially examined under acid or basic environmental medium. Generally, current laboratory methods for detecting FA include gas chromatography-mass spectrometry (GC-MS) [21], high-performance liquid chromatography (HPLC) [22,23,24], fluorimetry [25,26], and electrochemical sensors [27,28,29,30]. Electrochemical sensors, which can detect FA in both gas and aqueous forms, are considered as a striking method to detect FA. So far, various electrocatalysts, including noble metals, such as Pd, platinum (Pt) [27,31], gold (Au) [32,33], Pd-Au alloys [34], Pt-Pd NPs [35], Ag-Pd alloys [36,37], and supported catalysts, such as Au/CeO_2_ [38,39], Pd/TiO_2_ [40], and Co/In_2_O_3_ [41] catalysts, have been utilized to examine their electrochemical activities for FA oxidation in aqueous solution. Pd group metals based catalysts are the most frequently employed materials for electrochemical FA oxidation operated at acid or alkaline electrolytes. Moreover, attempts have been made to obtain catalysts with high surface area to achieve high efficiency of FA oxidation. Graphene, an atomic-layer-thick two-dimensional (2D) material, has become a star material, and has received intense attention from both experimental and theoretical points of view due to its versatile physical, chemical, electronic, mechanical, and thermal stability properties [42,43]. In addition to the unique electronic properties, the theoretically large surface area of 2600 m^2^ g^−1^ could endow graphene desirable characteristics as 2D support layers for metallic or bimetalic NPs in heterogeneous catalysis. As an allotrope of graphene, carbon nanotubes (CNTs) serve as support to prepare Pd/CNTs hybrids and used as electrochemical sensors for FA detection [44]. However, the weak response behavior of FA at different concentration could be detected and the results were rather unsatisfactory. Moreover, although Pd NPs [45], Pd-Pt bimetallic NPs [46], and Pd nanochains/WS_2_ nanosheets decorated graphene nanosheets [47] catalysts have shown preliminary advantages in FA detecting sensors, in the point view of practical application, they would still be hindered by high Pd loading, high overpotential, weak electrochemical response current, and a narrow electrochemical potential window of FA. Hence, there is incredible potential to develop versatile Pd based hybrids for FA detection, which possess excellent electrochemical response behaviors such as high sensitivity, a wide detection potential window, and lower overpotential by using lower Pd loading amount. Moreover, the interfacial interaction of metallic Pd species with graphene, as well as their fundamental roles in electrochemical detection of FA in aqueous solution, remains poorly understood and controversial.

For the reasons mentioned above, developing highly Pd-based catalysts and improving their hydrogen production efficiency and reducing the loading amount of Pd are of great significance for the large-scale application. In addition, it is urgent to develop highly efficient and cost-effectively functional electrocatalysts for FA detection. Herein, Pd NPs with different loadings supported on reduced graphene oxide (rGO) nanosheets catalysts were prepared by using the facile sodium borohydride reduction method. It was found that rGO nanosheets loaded with Pd NPs exhibited highly efficient catalytic activity in the hydrogen production from basic formaldehyde solution. The optimum reaction conditions and parameters, such as reaction temperature, NaOH concentration, FA concentration, and Pd loading, are achieved. Under the optimal reaction parameters, Pd/rGO with 0.5 wt% loading exhibited a hydrogen production rate of 272 mL g^−1^min^−1^, which is 3.2 times that of Pd NPs. Moreover, as-prepared Pd/rGO nanohybrids modified glassy carbon (GC) electrodes are used as FA-detected electrochemical sensors. The results showed that the prepared electrodes had good electrocatalytic properties with respect to formaldehyde electrooxidation as a result of the synergistic effect of rGO and Pd NPs combined with the GC technology. A sensitive oxidation peak with a current density of 8.38 mA/cm^2^ was observed at about 0.12 V (vs. Ag/AgCl) in 0.5 M NaOH containing 10 mM FA. In this paper, we highlight and expand the application of Pd/rGO composite catalysts with extremely low Pd loadings for efficient and stable catalytic hydrogen production from FA and electrochemical detection of FA in alkaline media.

## 2. Materials and Methods

### 2.1. Materials

Graphite powder (99%, Adamas), sulfuric acid (H_2_SO_4_, 95–98%, SCR), potassium permanganate (KMnO_4_, 99.5%, Greagent), sodium nitrate (NaNO_3_, ≥99%, Greagent), hydrogen peroxide (H_2_O_2_, ≥30%, Greagent), hydrochloric acid (HCl, 36–38%, SCR), sodium borohydride (NaBH_4_, 98%, SCR), palladium chloride (PdCl_2_, 99%, Pd: 59%, Adamas), sodium hydroxide (NaOH, 98%, Greagent), and formaldehyde (HCHO, 37–40% in water, Adamas) were used without further purification. Deionized water was used in all synthesis and reactions.

### 2.2. Preparation of the GO

Graphene oxide (GO) was synthesized by modified Hummers method [48]. First, 1 g of graphite powder and 0.5 g of NaNO_3_ were added to 23 mL of 98% H_2_SO_4_ in an ice bath environment. Then, 3 g of KMnO_4_ was added slowly under magnetic agitation. The mixture was stirred in an oil bath at 35 °C for 2 h. 40 mL of deionized water was added drop by drop and stirred for 30 min at 98 °C. Then 20 mL of H_2_O_2_ solution was slowly added to the mixture and stirred at 40 °C for 3 h. The mixture was washed several times with a HCl (1:10, V(HCl)/V(H_2_O)) solution and deionized water. The paste was evenly dispersed in 120 mL deionized water and dialyzed in a dialysis bag for 10 days. The collected sample was denoted as GO. 

### 2.3. Preparation of the Pd/rGO Catalysts

20 mL of GO was evenly dispersed in 40 mL of deionized water and 10 mL of ethanol. Then, a certain amount of PdCl_2_ solution containing 10 mL of 0.5 M of NaBH_4_ (freshly prepared) was added to the above solution drop by drop under stirring. The mixed solution was stirred continuously for 24 h at room temperature. The black solution was washed several times with ethanol and deionized water, and then freeze-dried for 12 h. The prepared samples were denoted as Pd/rGO-x% (x = 0.25, 0.5, 1, 3, which is corresponding to the theoretical mass ratio of Pd to GO). For comparison, Pd NPs were also prepared and the preparation process was the same as that of Pd/rGO, except that GO was not added in the synthesis procedure. Graphite powder (Aladdin, 99.95%, 8000 mesh) was also used as another carbon support to prepare Pd/C catalyst by impregnation-sodium borohydride reduction method (the theoretical loading of Pd was 0.5 wt%), the detail preparation procedure was the same as that of Pd/rGO.

### 2.4. Preparation of Nafion/GC and Nafion/GC-Pd/rGO Electrode

The bare GC electrode was polished with 0.03 μm alumina polishing powder on a micro-colth and then thoroughly cleaned with ethanol and distilled water several times. 2.0 mg of Pd/rGO powder was dispersed in 1.0 mL of 0.05% Nafion ethanol solution with ultrasonic treatment for 3 h to obtain a homogeneous suspension. Then, 5 μL of each homogeneous suspension was drop-casted on the surface of polished GC electrode for three times separately and dried at room temperature to obtain Nafion/GC-Pd/rGO electrode. The Nafion/GC was also prepared with the same procedure as that of Nafion/GC-Pd/rGO electrode except no GO was added.

### 2.5. Material Characterizations

Scanning electron microscopy (SEM) images were performed with a field emission scanning electron microscope Hitachi S-4800 (Tokyo, Japan). The microscopic characteristics of these materials were obtained at an acceleration voltage of 3 kV. The transmission electron microscopy (TEM) and high-resolution transmission electron microscopy (HRTEM) analysis were performed on a FEI Tecnai G2 F20 (Pittsburgh, PA, USA) field emission transmission electron microscope with a 200 kV accelerating voltage. The elemental composition of the samples was analyzed by the Thermo Scientific K-Alpha 0.5 EV (Thermo Fisher Scientific (China) Co., Ltd., Shanghai, China) X-ray photoelectron spectroscopy (XPS) using an Al Kα source. The powder X-ray diffraction (XRD) measurements were performed on a Rigaku Mini Flex 600 (Austin, TX, USA) diffractometer with a Cu Kα anticathode and the current of the lamp was 40 kV and 15 mA. X-ray patterns were collected in the 5–90° 2θ range with steps of 0.02°; the scan speed was 10°/min. The Fourier Transform Infrared (FTIR) was obtained from the Thermo Scientific Nicolet iS50 (Green Bay, WI, USA) spectrometer. Raman spectra were recorded by LabRAM HR Evolution (HORIBA France SAS, Longjumeau, French) with excitation wave number of 532 nm.

The electrochemical performance was performed with CHI660E electrochemical workstation (CH instruments, Shanghai, China) using three-electrode system. The modified glassy carbon (GC) electrode (3 mm diameter, CH instruments, Shanghai, China), Ag/AgCl electrode and platinum sheet were used as working electrode, reference electrode and auxiliary electrode, respectively. All electrochemical experiments were performed at ambient conditions.

### 2.6. Catalytic H_2_ production Performance

Specifically, 8 mg of catalyst was added to a glass bottle with an aqueous solution of sodium hydroxide at an appropriate concentration. The mixed solution was ultrasonic for 5 min to disperse the catalyst evenly, and then a certain amount of formaldehyde was added. The mouth of the bottle was quickly sealed and stirred, and the reaction began immediately. The hydrogen production reaction was initiated while the solution was violently stirred. Only H_2_ was analyzed through a gas chromatograph (GC) equipped with TCD detector and argon as carrier gas. During the reaction process, 150 µL of gas was extracted every 5 min and was quickly injected into the chromatographic inlet to analyze the evolved H_2_ gas. Only H_2_ and no other gas species were analyzed during the reaction. Each experiment was repeated three times to ensure the accuracy of hydrogen production. As for the cycling performance, after the first round of reaction, the catalyst was recovered by centrifugation and washing thoroughly with ethanol and distilled water several times, and then re-added to fresh formaldehyde solution.

## 3. Results

**Figure 1:**
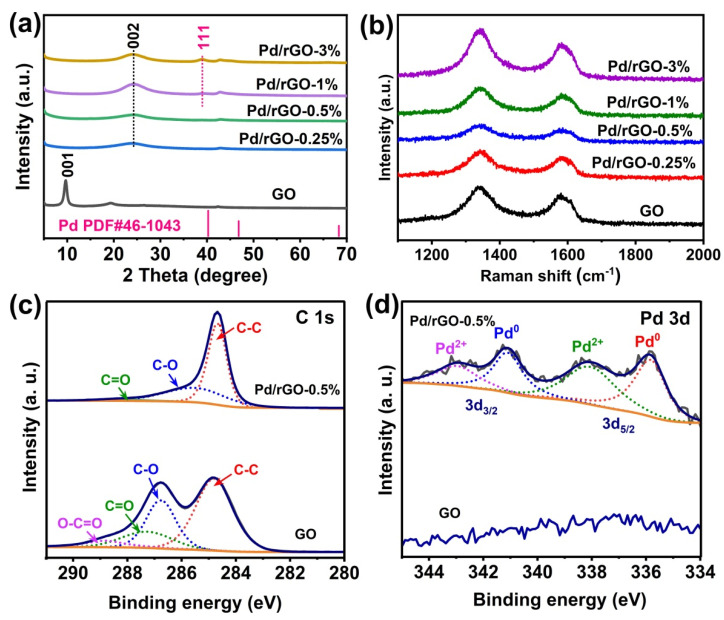
(**a**) XRD patterns and (**b**) Raman spectra of GO, Pd/rGO-0.25%, Pd/rGO-0.5%, Pd/rGO-1%, and Pd/rGO-3%; XPS spectra of (**c**) C 1s and (**d**) Pd 3d of the bare GO and Pd/rGO-0.5%.

Typical XRD patterns of the GO and Pd/rGO-x% samples are shown in Figure 1a. A strong and sharp peak at 2θ = 11.5° can be found in bare GO, which is related to the (002) basal plane of graphitic structure. Such a peak is also observed in the XRD spectra of Pd/rGO-x% but shifting to a higher 2θ value of 25°, which can be assigned to the characteristic structure of rGO that could be resulted from the formation of “re-graphitized” carbon regions and restacking due to the van der Waals interactions [49,50]. Meanwhile, the relative weak peak at 45° related to the (100) plane of graphitic structure can be also found for GO and Pd/rGO-x%, which is attributed to the partial unoxidized graphite. As for Pd/rGO-0.25% and Pd/rGO-0.5%, no obviously characteristic peaks of Pd species can be observed, which may be due to its low loading and good dispersion. In contrast, by further increasing the Pd loading to 1 wt% and 3%, the characteristic peak at 40.3°related to the (111) plane of metallic Pd species (PDF#46-1043) can be found for Pd/rGO-1% and Pd/rGO-3%, manifesting that Pd has successful loaded on the rGO support. To further confirm the existence of rGO, Raman spectra were performed. As shown in Figure 1b, two strong peaks were observed at 1345 and 1596 cm^−1^ for Pd/rGO-x% samples, which are attributed to the D and G bands of graphene, respectively. The D band and G band represent the disorder induced features caused by lattice defect and first-order scattering of the E_2g_ vibrational mode within aromatic carbon rings, respectively [51]. Raman spectroscopy shows that the intensity ratios of D band to G band for Pd/rGO-0.25%, Pd/rGO-0.5%, Pd/rGO-1%, Pd/rGO-3%, and Pd/rGO-5% are 1.22, 1.24, 1.38, 1.28, and 1.45, respectively. The structural defects, referred by D band, might serve as anchoring sites for Pd NPs and, subsequently, give rise to the restoration of sp^2^ network with small domains of aromatics [52,53]. XPS analyses show that the C 1s high-resolution spectra (Figure 1c) for bare GO can be deconvoluted into several peaks, corresponding to C-C bond (≈284.8 eV), oxygen containing functional groups of C-O (≈286.7 eV), C=O (≈287.4 eV), and O-C=O (≈288.9 eV) [54,55]. In contrast, in the presence of Pd species, most of the oxygen-containing functional groups for Pd/rGO-0.5% catalysts are removed following the NaBH_4_ reduction in the ethanol–water mixtures. Accordingly, the C 1s high-resolution spectra for Pd/rGO-0.5% can be deconvoluted into three peaks, corresponding to C-C (≈284.7 eV), C-O (≈285.8 eV) and C=O (≈287.8 eV) group. Moreover, the intensity ratio of the C-C to C-O peaks is found to increase from 1.07 in GO to 6.31 in Pd/rGO-0.5%. This indicates that the most oxygen-containing functional group can be reduced by NaBH_4_ in a mixed solution of ethanol and water. The unreduced part of oxygen-containing groups can be explained by the competition between Pd^2+^ and the sp^2^ regions of GO, which is consistent with the results of the reported literature [56]. Pd 3d XPS spectra in Figure 1d display two distinct peaks at 341.2 eV and 335.8 eV, corresponding to the metallic Pd^0^ 3d_3/2_ and Pd^0^ 3d_5/2_, respectively [56]. Additionally, two oxidation states are shown simultaneously at 342.9 and 338.1 eV, which are matched with the Pd^2+^ 3d_3/2_ and Pd^2+^ 3d_5/2_, respectively [57,58]. It is considered that the residual oxygen from rGO might react with Pd^2+^ ions since PdO is formed at low temperature. The N_2_ sorption curves of Pd/rGO-x% exhibit typical IV type isotherms with H_3_ hysteresis loop (Figure S1a). The large uptake at low relative pressure and distinct hysteresis loop between the adsorption and desorption branches implying the existence of both micropores and mesopores can be observed, which is consistent with the pore size distribution results (Figue S1b). The obtained Brunauer–Emmett–Teller (BET) specific surface area (S_BET_) is 149.97, 286.65, 302.43, and 352.97 m^2^g^−1^ for Pd/rGO-0.25%, Pd/rGO-0.5%, Pd/rGO-1%, and Pd/rGO-3%, respectively. For better and clear comparison, the structural information, including S_BET_, pore volume (V_p_), and average pore diameter (D_p_) for Pd/rGO catalysts, is summarized in Table S1.

**Figure 2:**
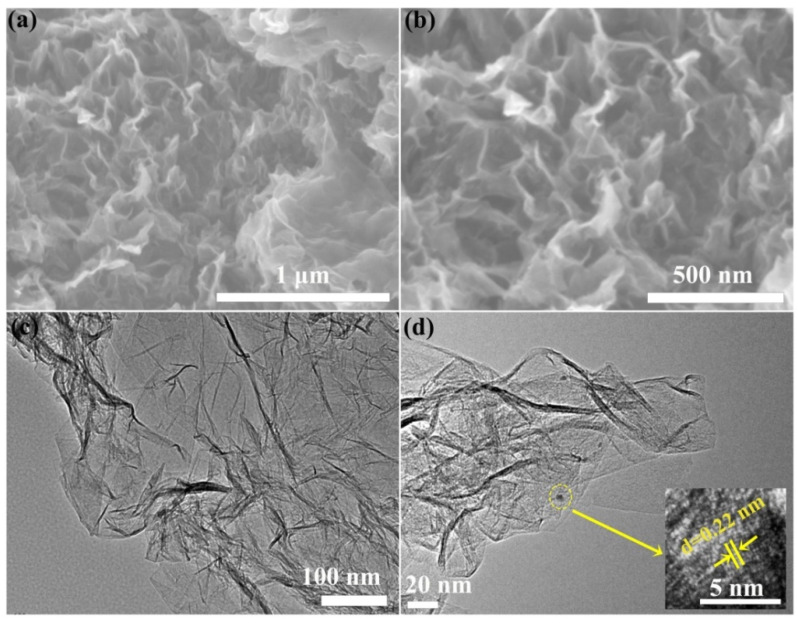
(**a**,**b**) SEM, (**c**) TEM and (**d**) HR-TEM images of Pd/rGO-0.5%. (The inset is enlarged view of the area circled in yellow of (**d**).)

The morphologies of as-prepared samples were thoroughly examined by SEM and TEM images. As shown in Figure S2a,b, GO exhibits typically planar nanosheets morphology with wrinkled and folded features. After reduction and loading with Pd NPs, the geometric morphology of GO is well preserved, as indicated by the SEM images shown in Figure S3 (Pd/rGO-0.25%), Figure S4 (Pd/rGO-1%), Figure S5 (Pd/rGO-3%), Figure 2a,b (Pd/rGO-0.5%), and TEM image shown in Figure 2c (Pd/rGO-0.25%). Due to the extremely low Pd loading (0.5 wt%), only very few Pd NPs can be observed from the HRTEM image shown in Figure 2d, which can be validated by the lattice fringes of Pd with 0.22 nm spacing (inset of Figure 2d). As for Pd/rGO-0.25%, the presence of Pd NPs was not observed on the TEM pictures shown in Figure S6a,b, probably due to the low loading of Pd species. In contrast, Pd NPs with ab average diameter of 20 nm can be observed when the loading of Pd is up to 1 wt% (Figure S7a). Meanwhile, as revealed by the HRTEM image in Figure S7b, the Pd NPs in Pd/rGO-1% sample has already exhibited partial agglomeration, which is unfavorable for the utilization of the active sites of Pd and the corresponding catalytic performance. As for Pd/rGO-3%, Pd NPs are the randomly dispersed and partial nanoparticles seem to aggregate to form larger cluster (>50 nm in diameter, Figure S8a,b).

**Figure 3:**
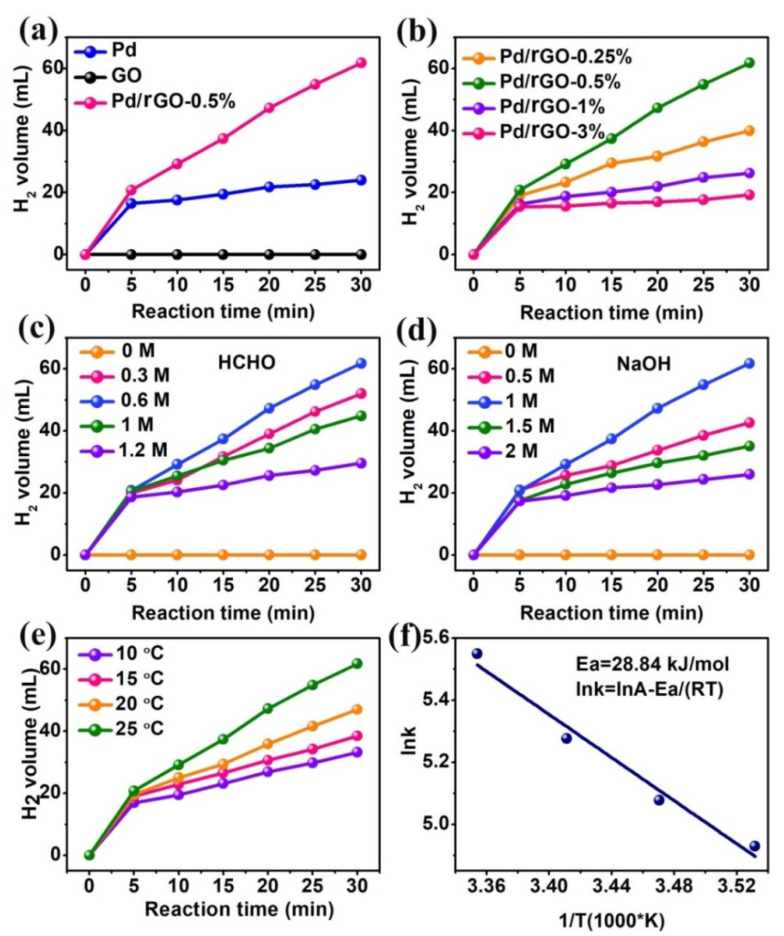
The effect of (**a**) catalysts and (**b**) Pd loading amount on the hydrogen generation. Reaction condition: T = 25 °C; NaOH: 1 M; HCHO: 0.6 M; catalysts: 8 mg. (**c**) The effect of HCHO concentration on the hydrogen generation. Reaction condition: T = 25 °C; NaOH: 1 M; catalysts: Pd/rGO-0.5%, 8 mg; (**d**) The effect of NaOH concentration on the hydrogen generation. Reaction condition: T = 25 °C; HCHO: 0.6 M; catalysts: Pd/GO-0.5%, 8 mg; (**e**) The effect of reaction temperature on the hydrogen generation. Reaction condition: HCHO: 0.6 M; NaOH: 1 M; catalysts: Pd/GO-0.5%, 8 mg. (**f**) Arrhenius plot (lnk versus 1/T) for the Pd/GO-0.5% catalyzed hydrogen production from HCHO solution.

The catalytic performance of the prepared GO and Pd/rGO-x% was systematically measured by evaluating their hydrogen production behavior in alkaline formaldehyde solution (Figure 3). Blank test with GO gives no traceable amount of hydrogen generation, indicating that pure GO is inactive for hydrogen evolution. The catalytic of H_2_ evolution increases rapidly after adding Pd/rGO-0.5% catalyst, 65.3 mL of H_2_ is generated in 30 min over Pd/rGO-0.5%, the corresponding datum over Pd NPs (typical TEM image is shown in Figure S9) is only 20 mL, indicating that Pd/rGO-0.5% catalyst exhibits higher activity than Pd NPs. The calculated hydrogen production rate over Pd/rGO-0.5% can reach 272 mL/min/g, which is 3.2 times that of conventional Pd NPs. Figure 3b shows the Pd loading effects on the hydrogen evolution of Pd/rGO-x% catalysts. With the increase of Pd loading, the hydrogen production of the catalyst first increased and then decreased. Specifically, the hydrogen production of Pd/rGO-0.25%, Pd/rGO-0.5%, Pd/rGO-1%, and Pd/rGO-3%, in 25 min was 10, 20, 30, and 40 mL, respectively, indicating that the optimal Pd loading in the current system was 0.5%. Figure 3c shows the HCHO concentration effects on the hydrogen evolution rate under room temperature of 25 °C. The hydrogen production increases from 0 mL to 52.5 mL with increasing FA concentration from 0 M to 0.3 M. Further increasing the formaldehyde concentration to 0.6 M leads to a maximum hydrogen production of 65.3 mL. However, further increasing the formaldehyde concentration to 1 M and 1.2 M, the hydrogen production volume drops dramatically to 48.1 and 30.3 mL, respectively. The above results show that in the current experimental system, the optimal concentration of formaldehyde is 0.6 M, and excessive formaldehyde will lead to the side Cannizzaro reaction, which is not conductive to the evolution of hydrogen. It is known that the PH of the reactant solution will seriously affect the hydrogen production performance of palladium-based catalysts, and an alkaline medium is necessary for formaldehyde conversion. Thus, the effect of NaOH concentration on the catalytic activity of Pd/rGO-0.5% for hydrogen generation was studied. As can be seen from results shown in Figure 3d, the highest hydrogen evolution rate can be achieved when the NaOH concentration is 1 M. As a promoter, NaOH can deprotonate the hydrated HCHO to form an active Cannizzaro intermediate (CH_2_(O^−^)_2_), which is a key step in the dehydrogenation of HCHO to produce formate and hydrogen (CH_2_(O^−^)_2_+H_2_O→HCOO^−^+H_2_+OH^−^) [9,59]. However, further increasing the NaOH concentration to 1.5 M and 2 M, the hydrogen production has been decreased obviously, which can be explained by the fact that too high concentration of NaOH will also lead to a side Cannizzaro reaction, which will also lower the hydrogen generation. Based on the above results, the optimal hydrogen production reaction conditions are: HCHO concentration: 0.6 M, NaOH concentration: 1 M, catalyst: Pd/rGO-0.5%. Figure 3e shows the effect of reaction temperature on the hydrogen generation from HCHO (0.6M)-containing solutions at 1 M NaOH over Pd/rGO-0.5% catalyst. It can be found that as the reaction temperature increases in a narrow range from 10 to 25 °C, the corresponding hydrogen production is linearly correlated to the reaction time at each temperature and increases from 32 to 65.3 mL, indicating that the reaction is extremely temperature-sensitive and conforms to the characteristics of first order reaction. Thereby, ln k versus 1/T is plotted in Figure 3f, combining the Arrhenius formula (k = Aexp (−E_a_/RT), where E_a_ is the apparent activation energy, k is the reaction rate constant, R is the molar gas constant, T is the absolute temperature), and the calculated activation energy is 28.84 kJ mol^−1^, which is much lower than the previously reported value of 65 kJ mol^−1^. 

**Figure 4:**
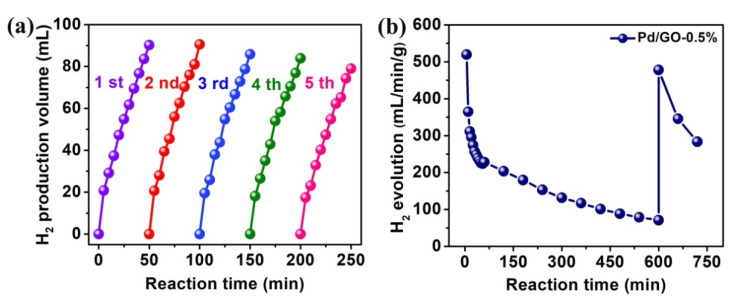
(**a**) Reusability and (**b**) long-term stability of H_2_ revolution over Pd/rGO-0.5% catalyst. Reaction condition: T = 25 °C; HCHO: 0.6 M; NaOH: 1 M; and catalysts: Pd/GO-0.5%, 8 mg.

Based on the above results, the prepared Pd/rGO-0.5% could be used as a highly efficient catalyst for hydrogen generation from alkaline formaldehyde solution at room temperature. However, for a catalyst with potential application, its efficient catalytic behavior is only one aspect, and another key point is the recyclability performance of the catalyst in long-term reactions. Hence, the reusability of Pd/rGO-0.5% over five cycles of catalytic hydrogen production is systematically, as revealed in Figure 4a. It can be found that after 5 cycles of catalytic reaction the hydrogen production does not decrease significantly, indicating that the prepared catalyst possesses excellent cycling and reusability performance. Moreover, the catalytic stability has been investigated under prolonged reaction time to 600 min as shown in Figure 4b, the initial hydrogen production rate can be reached as high as ca. 523 mL/min/g, but rapidly decreases to 256 mL/min/g after 60 min of reaction and then drops to 100 mL/min/g after 600 min of reaction. The reduction in the hydrogen production rate may be attibuted to the temporary poisoning of the catalyst and the dramatic reduction of reactant formaldehyde concentration, which can be validated by adding fresh formaldehyde solution after 600 min of reaction, and the hydrogen production rate can be restored to 500 mL/min/g. These results indicate that Pd/rGO could catalyze formaldehyde to produce hydrogen with an excellent reusability and stability in alkaline solutions.

**Figure 5:**
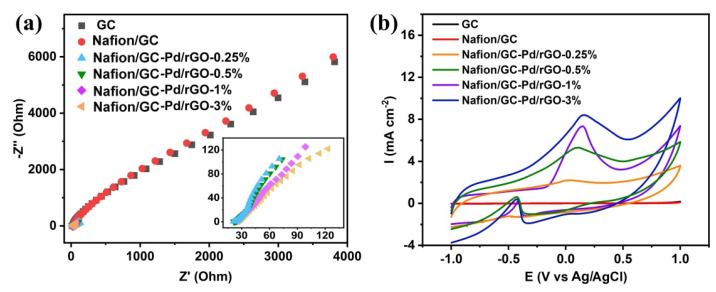
(**a**) Electrochemical impedance spectroscopy (EIS) of the bare GC electrode, the Nafion/GC electrode, the Nafion/GC-Pd/rGO-0.25%, 0.5%, 1% and 3% electrode in 10 mM formaldehyde solution containing 0.5 M NaOH. The frequency range was 10^5^ to 10^−1^ Hz at the bias voltage of 0.2 V. The inset is a larger view of Figure 5a in the low frequency region. (**b**) CVs curves of electrode, the bare GC electrode, the Nafion/GC electrode, the Nafion/GC-Pd/rGO-0.25%, 0.5%, 1%, and 3% electrode in 10 mM formaldehyde in 0.5 M NaOH at a scanning rate of 100 mV s^−1^.

**Scheme 1:**
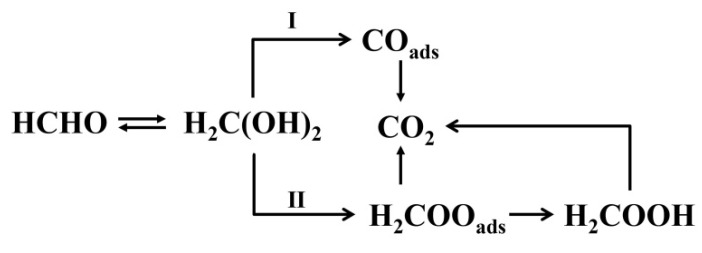
Schematic illustration of oxidation of formaldehyde.

In addition to evaluation of the hydrogen production performance of prepared catalysts from alkaline formaldehyde solution at room temperature, we prepared Pd/rGO catalysts modified GC electrodes used as formaldehyde-detected electrochemical sensors. Electrochemical AC impedance spectroscopy measured in 10 mM formaldehyde aqueous solution containing 0.5 M NaOH was examined to investigate the capability of electron transfer of modified electrodes. A well-defined semicircle at higher frequency can be found for bare GC electrode, indicating relatively high interface impedance. When Nafion is modified on the surface of bare GC electrode, the impedance value increases slightly, indicating that the introduction of Nafion makes the electron transfer at the interface more difficult. However, after further decorated with Pd/rGO catalyst, the semicircle decreases remarkably, which demonstrates that Pd-rGO hybrids can accelerate the electron transfer process on the interface of GC electrode. Such decreased impedance may be due to the excellent electrical conductivity and electron transport properties of Pd/rGO nanohybrids. Moreover, as shown in the inset of Figure 5a, the order of semicircle diameter, which reflects the charge-transfer resistance, is Nafion/GC-Pd/rGO-3% < Nafion/GC-Pd/rGO-1% < Nafion/GC-Pd/rGO-0.5% < Nafion/GC-Pd/rGO-25%. This indicates that the synergetic effect between Pd and rGO nanosheets and the higher loading of Pd are beneficial to improve the interfacial charge transport ability of the modified GC electrode. Moreover, cyclic voltammograms (CVs) of formaldehyde for bare and modified GC electrodes were obtained at a sweep rate of 100 mV/s in the voltage range of −1.0 to 1.0 V. As shown in Figure 5b, no response peaks are observed for both bare GC and Nafion/GC electrodes, indicating they have almost no electrochemical response to formaldehyde molecules. In contrast, two distinct peaks were observed at the modified GC electrodes. To be more specific, two oxidation peaks appear, respectively, at ca. 0.12 V and −0.43 V for Nafion/GC-Pd/rGO-0.25%, Nafion/GC-Pd/rGO-0.5%, Nafion/GC-Pd/rGO-1%, and Nafion/GC-Pd/rGO-3% electrodes. It is widely accepted that the oxidation mechanism of formaldehyde on Pd NPs modified electrode in an alkaline medium is a dual-path mechanism [34,60]. One pathway represents the indirect electrocatalytic oxidation of formaldehyde, which firstly forms a chemisorbed CO intermediate and eventually CO_2_ (pathway I, Scheme 1). The other pathway is the direct electrocatalytic oxidation process of formaldehyde, which forms CO_2_ through the formation of formaldehyde derivative intermediates (pathway II, Scheme 1). In the preferable reaction of path II, CO_2_ is directly formed through the formation of adsorbed active intermediates. In contrast, formaldehyde oxidation via an indirect path II produces adsorbed CO (CO_ads_), which acts as electrode fouling intermediate and, consequently, requires higher oxidation potential into CO_2_ [61,62]. Generally, in the whole process of electrochemical oxidation of formaldehyde, both the direct and indirect paths of formaldehyde oxidation can occur, which exhibits typically both forward (i_f_) and backward (i_b_) peak currents in the anodic zone. As for the modified GC-Nafion-Pd/rGO electrodes, the peak at ca. 0.12 V and −0.43 V can be attributed to i_f_ and i_b_, respectively_._ In addition, as shown in Figure 5b, i_b_ remains much smaller than i_f_, this can be explained by the preferred direct oxidation of formaldehyde catalyzed by Pd NPs on the modified GC electrode, further indicating that Pd NPs/rGO hybrids could provoke the reaction via direct pathway mechanism without formation of CO_ads_ intermediates. Moreover, the CVs results show that the i_f_ value increases with the increase of Pd loading, and the i_f_ value of Nafion/GC-Pd/rGO-0.25%, Nafion/GC-Pd/rGO-0.5%, Nafion/GC-Pd/rGO-1%, and Nafion/GC-Pd/rGO-3% electrode reaches 2.16, 5.15, 7.21, and 8.38 mA cm^−2^, respectively. Compared with bare and Nafion modified GC electrodes, the dramatically enhanced electrochemical detection capacity of formaldehyde for Nafion/GC-Pd/rGO-x% can be attributed to synergistic effects between the rGO and Pd NPs, i.e., Pd/rGO nanocomposites not only provides high surface area for formaldehyde accumulation, but also offers good electrocatalytic activity for oxidation of formaldehyde molecules. Meanwhile, rGO, as an excellent support material, can make Pd NPs highly dispersed, thus providing much more reactive sites. Density functional theory results reveal that Pd loaded single-walled carbon nanotubes (Pd/SWCNT) could be introduced as a better HCHO detector due to the electronic properties variation of Pd/SWCNT due to that the O atom in HCHO could prefer to be attached to Pd atom of Pd/SWCNT with the electronic charge transfer from HCHO to carbon nanotube rather than the H atom or C=O bond [63]. Moreover, it should be pointed out that although the higher the loading amount of Pd NPs, the higher the electrochemical response current of the prepared electrode to formaldehyde, but the higher loading of Pd means that the economic cost is also increased. Thus, in the following electrochemical formaldehyde detection process, Pd/rGO composites catalyst with a lower Pd loading was used.

**Figure 6:**
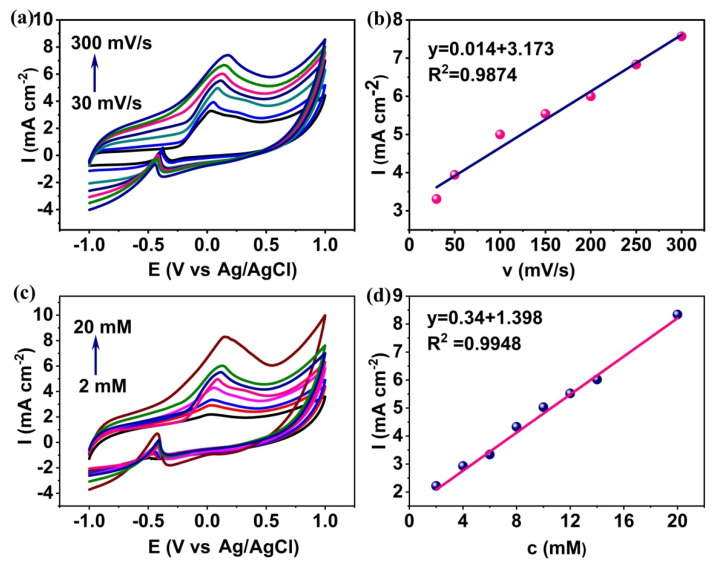
(**a**) CVs of Nafion/GC-Pd/rGO-0.5% electrode at different scan rates in 10 mM formaldehyde in 0.5 M NaOH. (**b**) Linear plots of the oxidation currents versus scan rates. (**c**) CVs of Nafion/GC-Pd/rGO-0.5% electrode at different concentration of formaldehyde (2 mM to 20 mM) rates in 0.5 M NaOH. (**d**) The relationship between oxidation current of formaldehyde and the concentration of formaldehyde.

The effects of formaldehyde concentration and CVs sweep rates on the kinetics of formaldehyde oxidation process over GC-Nafion-Pd/rGO-0.5% electrode were systematically investigated. The relation between the peak current and scan rate (v) is shown in Figure 6a. The linear relation between the peak current and scan rate (in the range of 30–300 mV s^−1^) indicates that the formaldehyde oxidation process is controlled by an adsorptive process. The peak current in the second scan is higher than that in the first scan, which further verifies the occurrence of adsorption process. Moreover, with the increase of scanning rate, the *i*_f_ value moves positively while the *i*_b_ value gradually shifts negatively. The related regression equation is: *i* (mA cm^−2^) = 0.014 + 3.173 V (mVs^−1^) (R^2^ = 0.9874) (Figure 6b). Figure 6c shows the dependence of the peak current obtained from CVs scan upon the addition of formaldehyde concentration from 2 mM to 20 mM over prepared Nafion/GC-Pd/rGO-0.5% electrode. As shown in Figure 6c, the anodic peak current is linearly related to the formaldehyde concentration in the range of 2 mM to 20 mM. The linear regression equation is *i* (mA cm^−2^) = 0.34 + 1.398 V (mVs^−1^) (R^2^ = 0.9948). 

Based on the above electrochemical measurements, it is found that the prepared Pd/rGO nanocomposites could serve as highly efficient electrocatalysts for the oxidation of formaldehyde. Although the detection limit temporary reaches only 2 mM under our current experimental conditions, the oxidation of formaldehyde reached at relatively low overpotential, which is more competitive in formaldehyde detection electrodes, such as platinum and palladium electrodes. Table 1 shows the comparison of the electrocatalytic behavior of as-prepared Pd/rGO for formaldehyde oxidation with some previously reported electrodes. The peak potential for the oxidation in our work is much less than that of reported electrodes. To the best of our knowledge, no electrochemical sensors for determination of formaldehyde using Pd or Pt based catalysts can be achieved at such a low overpotential and Pd loading amount. These results confirm the feasibility of the fabricated electrode for the quantitative detection of certain concentration of formaldehyde in aqueous solution. In order to highlight the excellent chemical reactivity of the prepared Pd/rGO samples, graphite powder was used as another carbon support to prepare Pd/C by the impregnation-sodium borohydride reduction method (the theoretical loading of Pd was 0.5 wt%), and evaluated its catalytic hydrogen production from formaldehyde and electrochemical formaldehyde detection performance, and the results are shown in Figure S10a,b. Under the same reaction conditions, although Pd/C exhibited higher hydrogen production activity than pure Pd NPs, its hydrogen production within 30 min was still much lower than that of Pd/rGO-0.5%. For the electrochemical detection performance of formaldehyde in alkaline medium, Nafion/GC-Pd/C electrode exhibited a current density of 2.68 mA cm^−2^, which was still much lower than that of Pd/rGO. In addition, its detection voltage was 0.34 V (V vs. Ag/AgCl), which was much higher than that of Pd/rGO. These results suggest that the Pd/rGO is a highly active catalyst for hydrogen production from formaldehyde as heterogeneous catalyst and detection of formaldehyde in alkaline medium as electrocatalyst.

## 4. Conclusions

The results presented here, for the first time that Pd NPs supported on reduced graphene nanosheets could serve as catalysts for efficient and stable hydrogen production from alkaline FA solution and electrocatalyst for electrochemical detection of FA in basic aqueous solution of FA. Under the optimized reaction conditions (FA concentration: 0.6 M, NaOH concentration: 1 M, reaction temperature: 25 °C), the Pd/rGO catalyst with 0.5 wt% Pd loading could exhibit a high production rate of 272 mL g^−1^ min^−1^, which is 3.2 times that of conventional Pd NPs. In addition, as-prepared Pd/rGO nanohybrids modified GC electrodes are used as FA-detected electrochemical sensors. The results showed that the prepared electrodes had good electrocatalytic properties with respect to formaldehyde electrooxidation as a result of the synergistic effect of rGO and Pd NPs combined with the GC electrode. The effects of formaldehyde concentration and CVs sweep rates on the kinetics of formaldehyde oxidation process over Nafion/GC-Pd/rGO-0.5% electrode were systematically investigated. A sensitive oxidation peak with a current density of 8.38 mA/cm^2^ was observed at 0.12 V (vs. Ag/AgCl) in 0.5 M NaOH solution containing 10 mM FA over Pd/rGO catalysts with Pd loading of 0.5 wt%. The results confirmed the feasibility of the prepared Pd/rGO catalysts for the highly and efficient hydrogen production from alkaline FA solution and quantitative detection of certain concentration ranges of FA in aqueous solution.

## Figures and Tables

**Table 1:** The comparison of electrochemical behavior for formaldehyde oxidation from the present work with previous reports.

Electrode	Electrolyte/Concentration of HCHO	Potential(V) (V vs. Ag/AgCl)
Pt/PAANI/MWNTs/GCE	0.5 M H_2_SO_4_/0.5 M	0.72 [64]
Pt(111) single crystal disk	0.1 M HClO_4_/0.5 M	0.70 [60]
Platinum modified SWNT/PANI composite	0.5 M HClO_4_/0.5 M	0.66 [65]
Pt-Pd/Ppy-CNT	0.5 M H_2_SO_4_/0.5 M	0.64 [62]
Pt–Pd/Nafion/GCE	0.1 M H_2_SO_4_/0.001 M	0.58 [35]
Ni-Pd/GCE	0.5 M NaOH/0.001M	0.43 [66]
Pt–SnO_2_/Ti	1 M HClO_4_/0.5 M	0.40 [67]
Pd-MWNTs	0.1 M NaOH/0.3 M	0.38 [44]
Pd-carbon ionic liquid composite	0.1 M NaOH/0.3 M	0.15 [68]
Nafion/GC-Pd/rGO (this work)	0.5 M NaOH/0.01 M	0.12

## Data Availability

Not applicable.

## Supplementary Materials

The following supporting information can be downloaded at: https://www.mdpi.com/article/10.3390/nano12111890/s1. Figure S1: N_2_ adsorption/desorption isotherms and pore size distribution; Table S1: Specific surface area, pore volume and average pore diameter results; Figure S2: TEM images; Figures S3–S5: SEM images; and Figures S6–S9: TEM images. Figure S10: Hydrogen production and electrochemical FA detection performance.
