# Supplementary material for: Highly Active Palladium-Decorated Reduced Graphene Oxides for Heterogeneous Catalysis and Electrocatalysis: Hydrogen Production from Formaldehyde and Electrochemical Formaldehyde Detection
Source: Nanomaterials (Basel). 2022 May 31;12(11):1890. doi: 10.3390/nano12111890 (PMC9182065; doi:10.3390/nano12111890)
Supplement: Supplementary file 1 [file nanomaterials-12-01890-s001.zip › nanomaterials-1750169-supplementary.pdf]

nanomaterials

Supporting Information

Highly Active Palladium-Decorated Reduced Graphene Oxides for Heterogeneous Catalysis and Electrocatalysis: Hydrogen Production from Formaldehyde and Electrochemical Formaldehyde Detection

Xiaogang Liu *, Wenjie Chen and Xin Zhang

College of Chemistry and Chemical Engineering, Xinyang Normal University, Xinyang 464000, China; chen2wen9jie5@163.com (W.C.); and zhangxin109zz@163.com (X.Z.)
* Correspondence: lxg133298@163.com

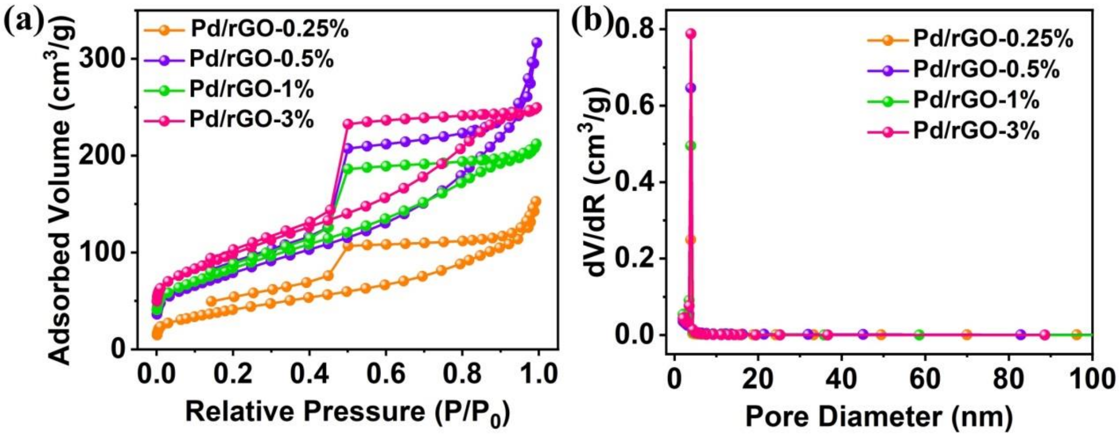

Figure S1. (**a**) N_2 adsorption/desorption isotherms and (**b**) the corresponding pore size distributions over prepared Pd/rGO-x% catalysts.

Table S1. Summary of the specific surface area (S_{BET}), pore volume (V_p) and average pore diameter (D_{ap}) for Pd/rGO-x% samples.

Samples	S_{BET} (m^2g^{-1})	V_p (cm^3g^{-1})	D_{ap} (nm)
Pd/rGO-0.25%	149.97	0.25	4.74
Pd/rGO-0.5%	286.85	0.54	5.24
Pd/rGO-1%	302.43	0.36	4.04
Pd/rGO-3%	352.97	0.43	4.25

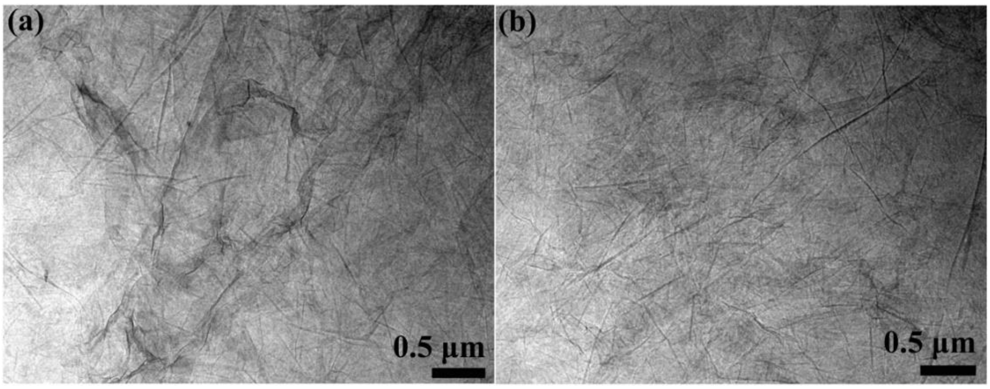

Figure S2. (**a**,**b**) TEM images of GO.

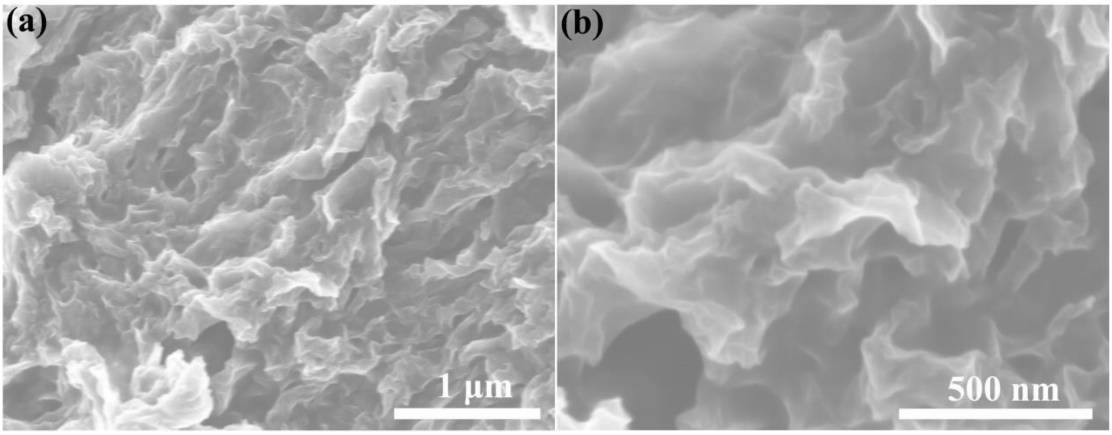

Figure S3. (**a**,**b**) SEM images of Pd/GO-0.25% with different magnification.

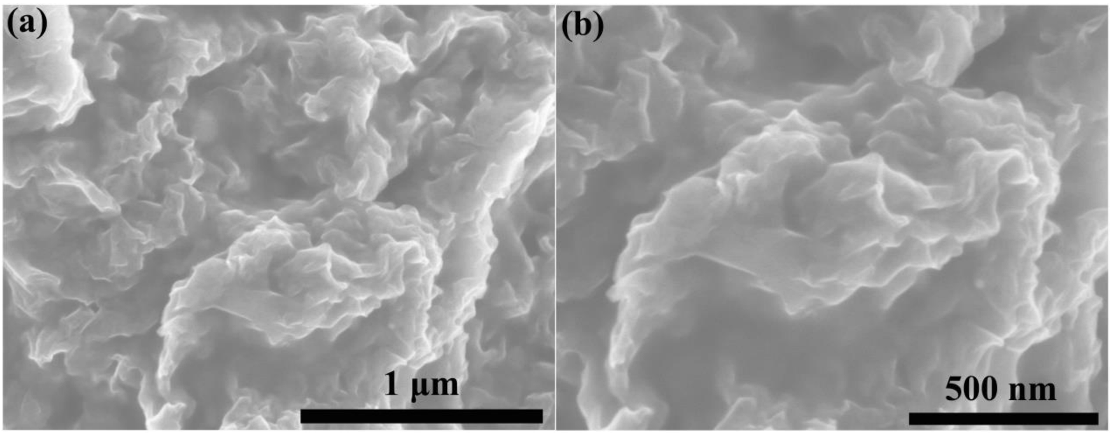

Figure S4. (**a**,**b**) SEM images of Pd/GO-1% with different magnification.

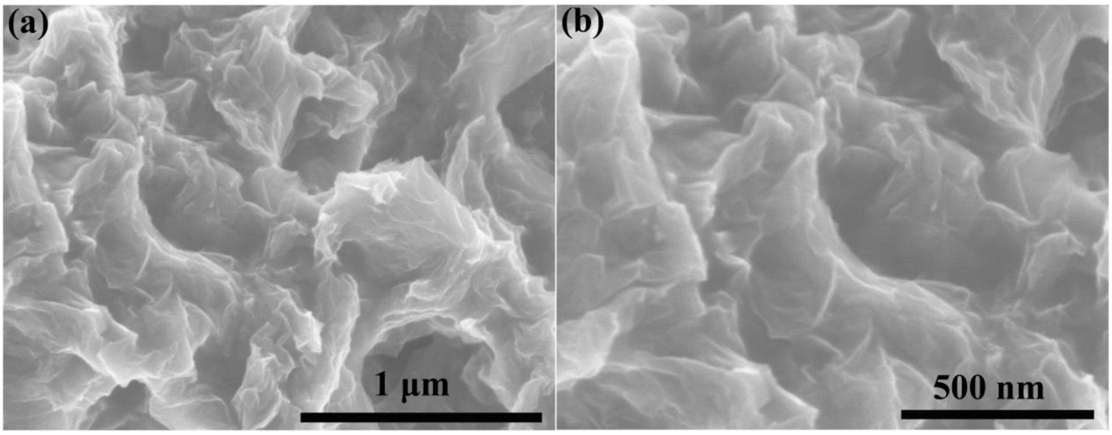

Figure S5. (**a**,**b**) SEM images of Pd/rGO-3% with different magnification.

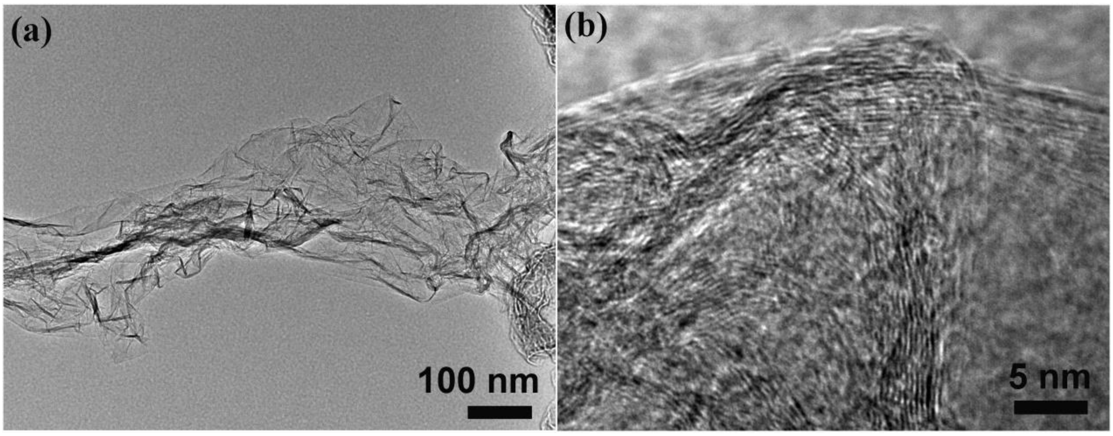

Figure S6. (**a**,**b**) TEM images of Pd/rGO-0.25% with different magnification.

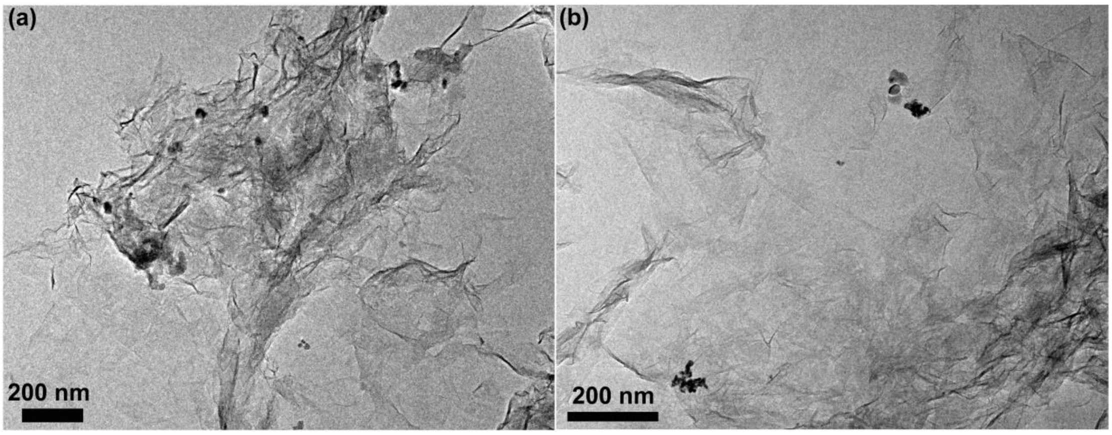

Figure S7. (**a**,**b**) TEM images of Pd/rGO-1% with different magnification.

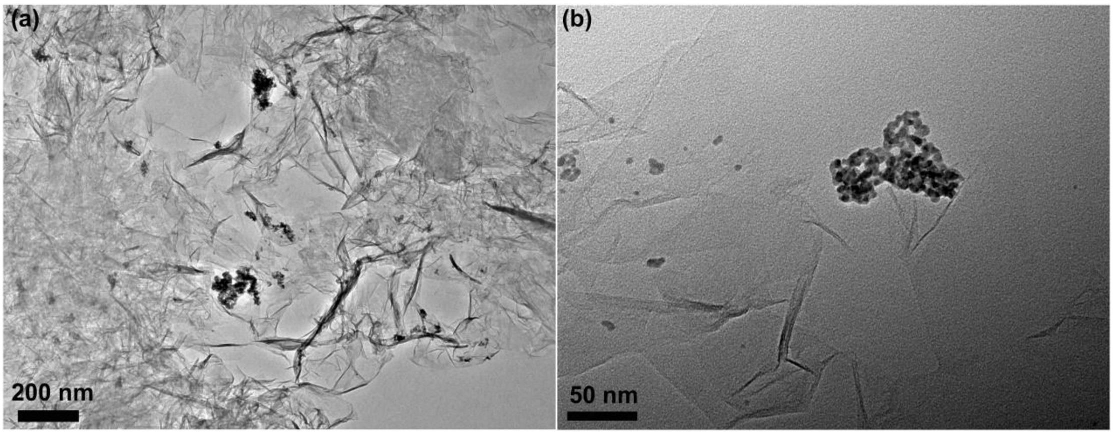

Figure S8. (**a**,**b**) TEM images of Pd/rGO-3% with different magnification.

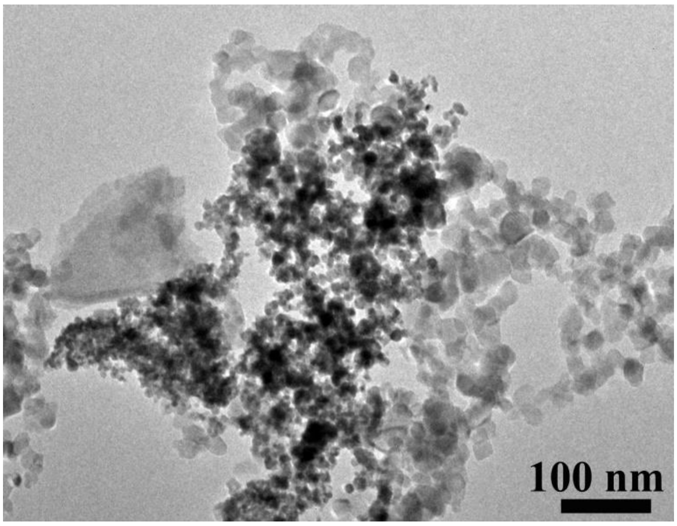

Figure S9. TEM image of prepared Pd NPs.

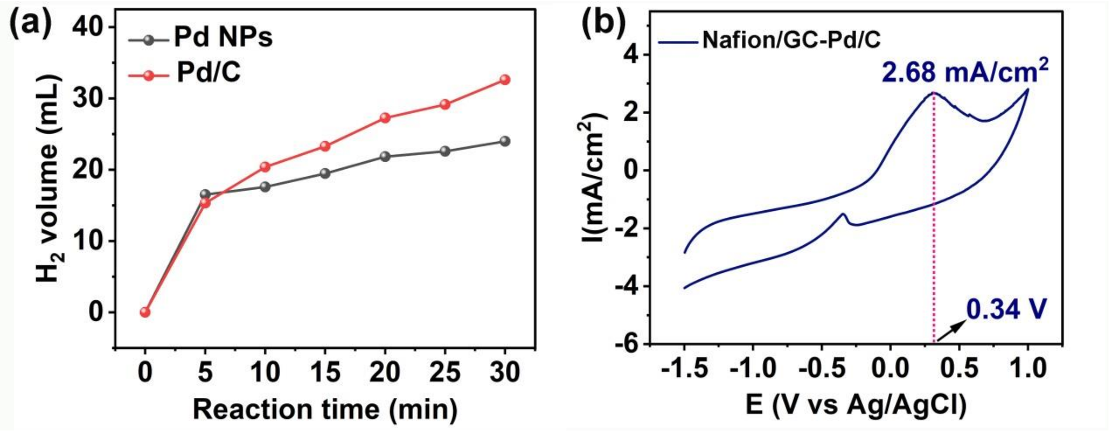

Figure S10. (**a**) H_2 production over Pd/C and Pd NPs. Reaction condition: T = 25 °C; HCHO concentration: 0.6 M; NaOH concentration: 1M; catalyst dosage: 8 mg. (**b**) The CV curves of the Nafion/GC-Pd/C electrode. Testing conditions: NaOH: 0.5 M; HCHO: 0.5 M; scanning rate:100 mV/s.
